# The Mouse Heart Mitochondria N Terminome Provides Insights into ClpXP-Mediated Proteolysis

**DOI:** 10.1074/mcp.RA120.002082

**Published:** 2020-11-23

**Authors:** Eduard Hofsetz, Fatih Demir, Karolina Szczepanowska, Alexandra Kukat, Jayachandran N. Kizhakkedathu, Aleksandra Trifunovic, Pitter F. Huesgen

**Affiliations:** 1 Institute for Mitochondrial Diseases and Aging at CECAD Research Centre, and Center for Molecular Medicine Cologne (CMMC), Medical Faculty, University of Cologne, Cologne, Germany; 2 Cologne Excellence Cluster on Cellular Stress Responses in Aging Associated Diseases (CECAD), Cologne, Germany, Medical Faculty and University Hospital, University of Cologne, Cologne, Germany; 3 Central Institute for Engineering, Electronics and Analytics, ZEA-3, Forschungszentrum Jülich, Germany; 4 Centre for Blood Research, School of Biomedical Engineering, Department of Pathology & Laboratory Medicine, Department of Chemistry, University of British Columbia, Vancouver, British Columbia, Canada; 5 Institute for Biochemistry, Faculty of Mathematics and Natural Sciences, University of Cologne, Cologne, Germany

**Keywords:** Proteolysis, degradomics, mitochondria function or biology, affinity proteomics, substrate identification

## Abstract

The mammalian mitochondrial proteome consists of more than 1100 annotated proteins and their proteostasis is regulated by only a few ATP-dependent protease complexes. Technical advances in protein mass spectrometry allowed for detailed description of the mitoproteome from different species and tissues and their changes under specific conditions. However, protease-substrate relations within mitochondria are still poorly understood. Here, we combined Terminal Amine Isotope Labeling of Substrates (TAILS) N termini profiling of heart mitochondria proteomes isolated from wild type and *Clpp*^−/−^ mice with a classical substrate-trapping screen using FLAG-tagged proteolytically active and inactive CLPP variants to identify new ClpXP substrates in mammalian mitochondria. Using TAILS, we identified N termini of more than 200 mitochondrial proteins. Expected N termini confirmed sequence determinants for mitochondrial targeting signal (MTS) cleavage and subsequent N-terminal processing after import, but the majority were protease-generated neo-N termini mapping to positions within the proteins. Quantitative comparison revealed widespread changes in protein processing patterns, including both strong increases or decreases in the abundance of specific neo-N termini, as well as an overall increase in the abundance of protease-generated neo-N termini in CLPP-deficient mitochondria that indicated altered mitochondrial proteostasis. Based on the combination of altered processing patterns, protein accumulation and stabilization in CLPP-deficient mice and interaction with CLPP, we identified OAT, HSPA9 and POLDIP2 and as novel bona fide ClpXP substrates. Finally, we propose that ClpXP participates in the cooperative degradation of UQCRC1. Together, our data provide the first landscape of the heart mitochondria N terminome and give further insights into regulatory and assisted proteolysis mediated by ClpXP.

## Teaser

We combined quantitative proteomics with N terminome profiling and substrate trapping AP-MS to identify new ClpXP substrates. Mitochondrial protein N termini isolated from mouse hearts revealed frequent aminopeptidase processing after MTS cleavage and many internal protease-generated neo-N termini. CLPP-deficient mice showed perturbed processing patterns with overall accumulation of protease-generated neo-N termini. New high-confidence candidate ClpXP substrates were identified by strong changes in N termini abundance or pull-downs with inactive CLPP and validated using immunoblotting and cycloheximide-chase experiments.

## Graphical Abstract

**Figure:**
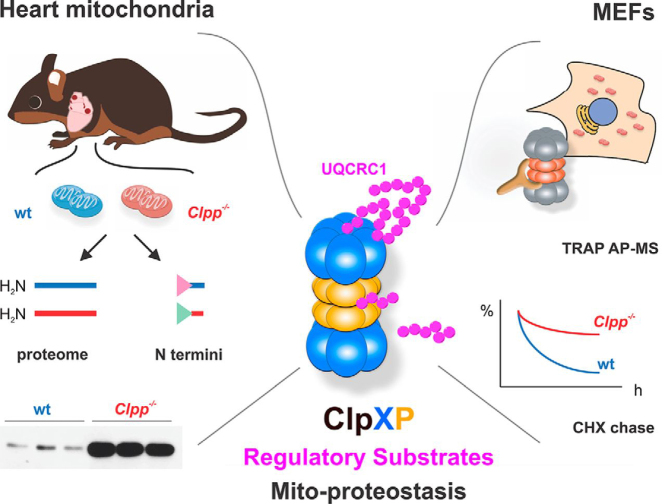

**Highlights**

• Mitochondrial heart N terminome shows aminopeptidase processing after MTS cleavage.
• CLPP-deficiency alters protein processing patterns in mouse heart mitochondria.
• Candidate substrates identified by N termini accumulation and interaction with inactive ClpXP.
• UQCRC1, HSPA9 and OAT validated biochemically as high confidence ClpXP substrates.

Mitochondria are essential organelles for almost all eukaryotic cells; thus, sustaining the integrity of their proteins is crucial for cell viability. Given that the proteome is of dual origin, mitochondria face the difficulty of coordinating protein synthesis, import, and maintenance. Because of the high mitochondrial compartmentalization, soluble proteins in the matrix or the intermembrane space, as well as hydrophobic proteins in the inner or outer membrane, must be processed independently. Mitochondria have several levels of protein quality control operating on the molecular or cellular level. As a result of the alphaproteobacterial origin of mitochondria, most of the proteolytic quality control system shares strong similarity between mitochondria and today's proteobacteria, such as *Escherichia coli*. Proteolytic control within mitochondria is predominantly performed by ATP-dependent proteases, which include the soluble ClpXP and LONP1 proteases in the mitochondrial matrix and membrane-bound i-AAA and m-AAA proteases that face the intermembrane space and matrix, respectively (1). Whereas LONP1 is mainly attributed with quality control of misfolded (2) and oxidatively damaged proteins (3), or mtDNA maintenance via the proteolytic control of mitochondrial transcription factor A (TFAM) (4), relatively little is known about substrates and physiological relevance of the caseinolytic peptidase ClpXP (1). In *E. coli*, up to 60% of all protein degradation seems to be ATP-dependent proteolysis executed by the Lon and ClpXP/ClpAP proteases (5). Both proteases have specific regulatory substrates as well as housekeeping functions, and it seems that also in bacteria, most unfolded or damaged proteins are degraded by the Lon protease (5). Remarkably, tightly folded substrate domains can result in stalling of processive degradation by ClpP in bacteria, resulting in the release of processed proteoforms. A prominent example is the partial N-terminal proteolysis of DnaX by *Caulobacter crescentus* ClpXP, which generated distinct DnaX proteoforms that are important to support normal growth (6).

The functional ClpXP protease consists of the protease subunit CLPP (Caseinolytic Peptidase ATP-dependent, Proteolytic Subunit), and the ATP-dependent chaperone CLPX (7). In the presence of CLPX and ATP, the two CLPP heptameric rings form the catalytic tetradecamer chamber with 14 serine/histidine/aspartate triads that catalyze peptide cleavage (8). The chaperone CLPX forms hexameric rings that stack on one or both sides of the CLPP chamber to allow ATP-dependent protein unfolding and translocation through the axial channel into the catalytic core (9, 10). In the absence of CLPX unfoldase, which also ensures the identification of specific substrates, CLPP is still able to cleave small, unspecific peptides (10). The 3-dimensional structure and biochemical properties of ClpXP have been well described in bacteria and are largely conserved in eukaryotes (7, 8). However, substrates, specific adaptors, and the function of mitochondrial ClpXP remain mostly enigmatic.

We have recently shown that the whole-body CLPP knockout mice (*Clpp*^−/−^) are sterile with shorter stature, as observed in Perrault syndrome patients (11, 12). Remarkably, they live a normal life-span with improved glucose metabolism that renders them resistant to diet-induced obesity (11). Particularly in the heart, mammalian CLPP is an important regulator of mitochondrial protein synthesis through the removal of ERAL1 from the small ribosomal subunits to allow assembly of the functional mitoribosome (13). Contrary to nematodes, CLPP appears not to be needed for the activation or maintenance of the mammalian mitochondrial unfolded protein response (UPR^mt^) (14). Unexpectedly, loss of CLPP alleviates the strong mitochondrial cardiomyopathy and partially restores the diminished respiration in animals with a strong dysregulation of mitochondrial protein synthesis (14). Together, these results question our current understanding of ClpXP-mediated proteolysis in mammals, while introducing CLPP as a possible novel target for therapeutic intervention in mitochondrial diseases (14).

Mass spectrometry-based proteomics has become the predominant tool for unbiased characterization of protease substrates and function (15). Quantitative proteomics can reveal candidate substrates of degradative enzymes, including ClpXP, based on the change in abundance between different genotypes and treatments (13), whereas the combination with affinity enrichment of native or epitope-tagged active and inactive versions of a protease can identify interaction partners, regulatory proteins and substrates (13, 15). In addition, a variety of protocols for selective enrichment, identification and quantification of protein amino (N)- or carboxyl (C)-terminal peptides (16, 17) enable unbiased identification of processing patterns and direct determination of protease substrates with their precise cleavage sites (18, 19). Global profiling of mitochondrial protein N termini was used to define the MTS cleavage sites (20, 21), revealed subsequent processing by the aminopeptidases ICP55 and OCT1 (21, 22, 23) and identified the proapoptotic protein Smac/DIABLO as a substrate of the mitochondrial rhomboid protease PARL (24). N termini profiling in mitochondria isolated from human cells has further been applied to characterize protein processing early after activation of the intrinsic apoptosis pathway (25) and in response to zinc and rapamycin treatment (26).

Here, we combined two mass spectrometry-based approaches to investigate the impact of CLPP on the mitoproteome and systematically identify putative ClpXP substrates. Profiling of protein N termini in control and CLPP-deficient heart mitochondria by Terminal Amine Isotope Labeling of Substrates (TAILS) (27) revealed altered cleavages in accumulating candidate ClpXP substrates, including OAT and HSPA9, as well as moderate but widespread alterations in mitochondrial protein processing. In addition, we identified putative substrates by expressing FLAG-tagged, proteolytically active and inactive versions of CLPP in *Clpp*^−/−^ mouse embryonic fibroblasts (MEFs), followed by affinity purification and mass spectrometric quantification (9). Using this approach, we identified a small subset of specifically trapped mitochondrial matrix proteins including UQCRC1, POLDIP2, and NDUFV2 as candidate substrates. Cycloheximide-chase demonstrated stabilization of HSPA9, OAT, and UQCRC1 in *Clpp*^−/−^ MEFs, validating these proteins as novel bona fide ClpXP substrates. Taken together, our results provide a first large-scale experimental characterization of protein maturation and processing in heart mitochondria and support the hypothesis that mammalian ClpXP primarily functions as a proteolytic regulator of specific substrates, but also directly or indirectly contributes to mitochondrial protein quality control.

## EXPERIMENTAL PROCEDURES

##### Mouse Strains and Ethics

*Clpp*^−/−^ mice were generated, maintained and genotyped as previously described (13). 17-weeks-old animals were used for the TAILS analysis. All experiments were approved and permitted by the Animal Ethics Committee of North-Rhein-Westphalia (Landesamt für Natur, Umwelt und Verbraucherschutz Nordrhein-Westfalen; LANUV) following German and European Union regulations. All animal work was performed in accordance with recommendations and guidelines of the Federation of European Laboratory Animal Science Associations (FELASA).

##### Mitochondrial Preparations from Hearts

Mitochondria were isolated from wild type and *Clpp*^−/−^ hearts at the age of 17 weeks as previously described (13). In short, freshly isolated hearts were homogenized with a Teflon homogenizer in mitochondria isolation buffer (100 mm sucrose, 50 mm KCl, 1 mm EDTA, 20 mm TES, pH 7.2) with the addition of subtilisin A (1 mg/g heart) and 0.2% BSA (fatty acid free). Crude mitochondrial fractions were obtained through differential centrifugation at 8500 × *g*, 800 × *g*, and again at 8500 × *g* to remove fat, heavy fractions, and to pellet mitochondria, respectively. The mitochondrial pellet was washed with mitochondria isolation buffer without subtilisin A/BSA and either stored at −80 °C for Western blot analysis in the same buffer or resuspended in 6 m GuHCl for TAILS experiments.

##### Cell Lines

Wild type and *Clpp*^−/−^ MEFs were isolated, immortalized and cultured in high glucose DMEM at 37 °C and 5% CO_2_ as previously described (28).

##### CLPP Substrate Trapping in MEFs

Three independent *Clpp*^−/−^ MEF lines were transfected with each empty vector (NEG), CLPP-WT-FLAG (WT) and a catalytically inactive version with Ser149 mutated to Ala, CLPP-TRAP-FLAG (TRAP) (13) using the Nucleofector (Lonza, Basel, Switzerland) electroporation kit according to the manufacturer instruction. 72h after transfection, 80% confluent cells were harvested with trypsin, washed twice with PBS and lysed for 45 min in 300 μl IP buffer (Thermo Fisher Scientific, Schwerte). Samples were incubated with 30 μl α-FLAG magnetic beads (Sigma) over night at 4 °C on a rotating wheel. On the following day, beads were washed 4 times with IP buffer and bound proteins were eluted in 70 μl Elution buffer (Thermo Fisher Scientific). Samples were neutralized with 10 μl 1 m Tris/HCl pH 7.5, snap-frozen and stored at −80 °C. 1% of each Lysate (L), unbound proteins (F), first washing solution (W) and 10% of the Elution fractions (E) were used for SDS-PAGE controls. Samples were prepared for proteomic analysis as previously described (13).

##### Cycloheximide (CHX) Chase Experiments

**Fig. 7:**
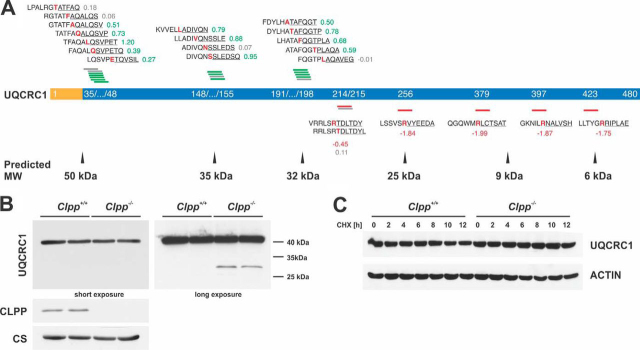
**Validation of UQCRC1 as an unexpected ClpXP substrate.***A*, UQCRC1 N termini abundance. MTS (yellow) and mature protein (blue) are shown with starting position of detected N termini. Above and below the protein, identified N-terminal peptides (underlined) are shown with the associated log_2_(*Clpp*^−/−^/wt) and the preceding sequence. Amino acids at the P1 position are highlighted in red. N termini with increased abundance are depicted in green, with decreased abundance in red and unchanged abundance in gray. Predicted masses of the corresponding proteoforms are indicated below the scheme. *B*, Western blots of UQCRC1 steady state levels in isolated mitochondria. Citrate synthase (CS) was used as loading control. *C*, CHX chase experiment of UQCRC1 in MEFs. ACTIN was used as loading control (13).

CHX chase experiments were performed as previously described (13) and Western blotting membranes were incubated with antibodies against newly identified candidate substrates. For Fig. 7*C*, the published loading control (ACTIN) for UQCRC1 was reused as the same membrane was probed with anti-UQCRC1 (13). New CHX chase experiments were performed for OAT and HSPA9. All antibodies used in this study are listed in Table I.

**Table I:** Antibodies, manufacturer and used conditions

Antibody	Manufacturer	Cat. Nr.	Conditions
CLPP	Sigma	HPA040262	1:1000 (5% Milk PBST)
CLPX	Sigma	HPA040262	1:1000 (5% Milk PBST)
C1QBP/P32	Millipore	AB2991	1:1000 (5% Milk PBST)
UQCRC1	Molecular Probes	459140	1:2000 (5% Milk PBST)
HSPA9	Abcam	82591	1:1000 (5% Milk PBST)
LONP1	Abcam	ab82591	1:1000 (5% Milk PBST)
OAT	Abcam	ab137679	1:1000 (5% Milk PBST)
CALNEXIN	Calbiochem	208880	1:1000 (5% Milk PBST)
β-ACTIN	Sigma	A5441	1:5000 (5% Milk PBST)
HSC70	SantaCruz	sc-7298	1:5000 (5% Milk PBST)
POLDIP2	CUSABIO	CSB-PA896496LA01HU	1:2000 (5% Milk PBST)
TOMM20	SantaCruz	sc-17764	1:2000 (5% Milk PBST)
FLAG M2	Sigma	F1804	1:1000 (5% Milk PBST)

##### RNA Isolation and qPCR

Isolated heart RNA was treated with DNase (DNA-free Kit, Thermo Fisher Scientific) and subsequently reverse transcribed with the High-Capacity cDNA Reverse Transcription Kit (Thermo Fisher Scientific). Gene expression levels were determined with qPCR technique using Brilliant III Ultra-Fast SYBR Green qPCR Master Mix (Agilent Technologies, Waldbronn, Germany). Samples were adjusted for total RNA content by Hypoxanthine-guanine phosphoribosyltransferase (*Hprt*). Primers used are listed in Table II.

**Table II:** Primers used for qPCR

Gene	FWD 5′-3′	REV 5′-3′
*Hspa9*	ATGGCTGGAATGGCCTTAGC	ACCCAAATCAATACCAACCACTG
*Lonp1*	ATGACCGTCCCGGATGTGT	CCTCCACGATCTTGATAAAGCG
*Oat*	GGAGTCCACACCTCAGTCG	CCACATCCCACATATAAATGCCT
*Hprt*	GCCCCAAAATGGTTAAGGTT	TTGCGCTCATCTTAGGCTTT

##### N Terminome Profiling

N-terminal peptides were enriched from isolated heart mitochondria using TAILS (Terminal Amine Isotope Labeling of Substrates) with differential formaldehyde isotopes (^12^CH_2_O and ^13^CD_2_O) and sodium cyanoborohydride (NaBH_3_CN) for modification and labeling of primary amines as described (29). The following labeling scheme was used: control, light label (^12^CH_2_O); *Clpp*^−/−^ mutant, heavy label (^13^CD_2_O). Labeled samples were digested with trypsin (Serva, Electrophoresis, Heidelberg, Germany) overnight, then a small aliquot of ∼10 μg proteome was withdrawn to control for digestion efficiency by SDS-page analysis and labeling efficiency by shotgun proteome analysis (designated “preTAILS” samples). For enrichment of N-terminal peptides, free trypsin-generated primary amines were subsequently coupled to HMW-ALD polymer and internal peptides bound to the polymer removed by ultrafiltration, leaving enriched N-terminal peptides as “TAILS” samples in the flow-through. All peptides were desalted using in-house packed C18 StageTips (28) and additionally crudely fractionated (15–20-30–50% ACN) at neutral pH in 20 mm ammonium bicarbonate buffer (AmBiC) using SepPak C18 cartridges (Waters, Eschborn, Germany). Samples were analyzed using an Ultimate 3000 RSLCnano HPLC (Thermo, Germering, Germany) operated in a two-column setup (Acclaim PepMap 100 C18, particle size 3 μm, ID 75 μm for trap and ID 50 μm for analytical column; trap column length 2 cm, analytical column length 25 cm, Thermo) at a flow rate of 350 ml/min at 60 °C. The nano LC system was on-line coupled to an Impact II high resolution Q-TOF (Bruker, Bremen, Germany) via a CaptiveSpray nano ESI source (Bruker) with a NanoBooster (Bruker) engaged to saturate the nitrogen gas stream with acetonitrile essentially as described (30).

##### Mass Spectrometry Data Analysis

For analysis of preTAILS and TAILS samples, spectra were matched to peptide sequences at a FDR of 0.01 using MaxQuant (31), v.1.6.0.16 with the UniProt mouse proteome (canonical + isoforms, release 2017_10, 60717 entries) as a database for searches with standard Bruker QTOF instrument settings, which included 0.07 Da MS precursor tolerance in the first search, 0.006 Da precursor mass tolerance in the second search after recalibration, and MS/MS spectra matching tolerance of 40 ppm. Mass spectra acquired from preTAILS samples were searched twice: To control efficiency of the different labeling steps, searches considered trypsin as digestion enzyme allowing for up to four missed cleavages, Cys carbamidomethylation was set as fixed modification, and isotopically light (+28.031300) and heavy (+36.075670) dimethylation of Lys residues and Met oxidation as variable modifications. This search confirmed that >98% of the identified Lys residues were dimethyl labeled and < 6% of the identified peptides were cleaved after dimethylated Lys residues, in agreement with inefficient trypsin activity toward dimethyl modified Lys (27, 32). Therefore, for analysis of protein abundance changes, a second analysis was performed considering ArgC as digestion enzyme, duplex isotope labeling by light (+28.031300) and/or heavy (+36.075670) dimethylation of Lys residues, Cys carbamidomethylation as fixed and Met oxidation as variable modification (33). Search parameters for N termini identification and quantification were set to semi-specific (free N terminus) ArgC as digestion enzyme, isotope labeling by light (+28.031300) and/or heavy (+36.075670) dimethylation of Lys residues and peptide N termini, Cys carbamidomethylation as fixed and Met oxidation, N-terminal acetylation (+42.010565) or N-terminal pyroGlu formation from Glu (-18.010565) or Gln (-17.026549) as variable modifications. The “requantify” and “match between runs” functions were enabled. False discovery rates, estimated using an appended reverse-decoy protein database, were set to 0.01 at PSM and protein levels. For preTAILS samples, MaxQuant protein ratios were recalculated considering only matching Lys-containing quantified peptides. For TAILS data, identified N-terminal peptides were annotated based on the modificationSpecificPeptides.txt file of the MaxQuant output folder with information from Uniprot.org and sequence windows using an in-house script (MaxQuant Advanced N Termini Interpreter, MANTI.pl version 3.7, https://sourceforge.net/projects/manti/). Pyro-Glu modified peptides may arise during sample preparation after tryptic digest and were therefore excluded from further analysis, as were peptide identifications that did not contain a C-terminal Arg or reported with a nonsense dimethyl modification at N-terminal Pro. Dimethylated or acetylated peptides matching to positions 1 or 2 of a protein model or termini starting within 5 amino acid window deviation from UniProt-annotated MTS cleavage sites were defined as “expected” termini, whereas all peptides matching to positions within the protein model were considered “unexpected” protein N termini, which include mostly protease-generated neo-N termini but also unannotated proteoforms arising from alternative splicing or use of alternative translation initiation sites (19). Further data analysis was performed with Perseus version 1.6.6 (34).

For the CLPP-TRAP experiment, spectra were matched to peptide sequences using MaxQuant v1.5.3 (31). using the UniProt Mouse protein database (release 2015_01, 44654 entries) and standard settings for the QExactive instrument, Thermo Scientific, Bremen, Germany, including 20 ppm MS precursor tolerance in the first search, 4.5 ppm precursor mass tolerance in the second search after recalibration, and MS/MS spectra matching tolerance of 20 ppm. Enzyme specificity set to trypsin with two missed cleavages allowed, cys alkylation with iodoacetamide was set as fixed and Met oxidation as variable modifications. Label free quantification and match between runs was enabled with preset standard settings. False discovery rates, estimated using an appended reverse-decoy protein database, were set to 0.01 at PSM and protein levels. Further data analysis was performed with Perseus version 1.6.6 (34). Proportional Venn diagrams were drawn with the BioVenn web application (35).

##### Experimental Design and Statistical Rationale

All experiments were performed in biological triplicates for each experimental condition. In the trapping experiment, proteins were tested for differential abundance between CLPP-FLAG, CLPP-TRAP-FLAG and control conditions by multi-sample ANOVA as implemented in Perseus, v.1.6.6 (34). Differences associated with Benjamini-Hochberg corrected ANOVA q-value <0.05 were considered significant. Quantitative analysis of the N-terminal peptides considered only peptides quantified in at least 2 out of the 3 biological replicates. The significance of the difference in the median abundance between expected and unexpected protein N termini were tested by Mann-Whitney *U* test. Additionally, a cut-off for strong changes in N termini abundance was defined based on the boxplot analysis of 120 N termini mapping to proteins that showed no significant change in abundance in a previous label-free LC-MS proteome analysis (13).

## RESULTS

##### Profiling of Mitochondrial Protein N Termini in Wild Type and CLPP-deficient Mouse Hearts

**Fig. 1:**
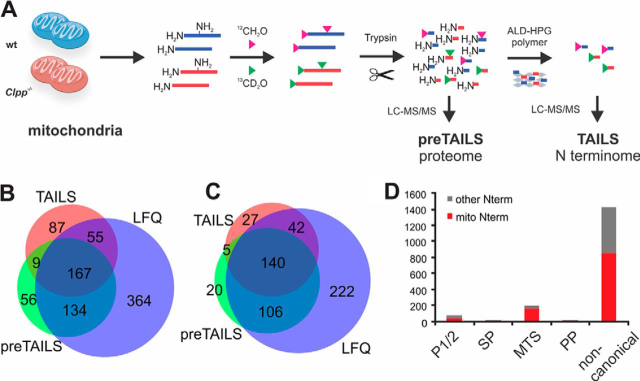
**Mouse heart N terminome profiling.***A*, Scheme of the TAILS workflow. Protein N termini and Lys side chains from wild type (wt) and *Clpp*^−/−^ mitochondrial proteins were labeled with light and heavy formaldehyde, respectively, pooled and digested with trypsin. A “preTAILS” aliquot was withdrawn for labeling control and determination of protein abundance changes. In the next reaction, peptides with unlabeled N termini resulting from tryptic digestion were covalently captured with a high-molecular weight aldehyde-functionalized ALD-HPG polymer. Removal of the polymer with bound peptides by ultrafiltration left labeled N-terminal peptides highly enriched in the flow-through for LC-MS/MS analysis. *B*, Overlap of all proteins identified by the preTAILS and TAILS in this analysis and our previous LFQ proteome analysis (13). *C*, Overlap of proteins with UniProt-annotated mitochondrial localization between preTAILS, TAILS and LFQ data sets. *D*, Positional annotation classifying protein N-terminal peptides identified after TAILS enrichment into 5 categories, those matching position 1 or 2 of the protein model, matching within 5 amino acids of annotated or predicted signal peptide (SP), mitochondrial targeting signal (MTS) cleavage sites, propeptide maturation sites (PP) or those matching to “unexpected” positions within the protein model. Red bars indicate proteins with mitochondrial location as annotated by UniProt.

To gain insights into mitochondrial protein processing in CLPP-deficient mice, we first isolated mitochondria from 17 weeks-old wild type and *Clpp*^−/−^ hearts, extracted proteomes under denaturing conditions and enriched N-terminal peptides by Terminal Amine Isotope Labeling of Substrates (27). In brief, primary amines at protein N termini and Lys side chains in wild type and *Clpp*^−/−^ mitoproteomes were differentially labeled with stable isotopes (light and heavy formaldehyde, respectively), before trypsin digestion (Fig. 1*A*). A small “preTAILS” sample was withdrawn after digestion and before N termini enrichment to control dimethyl labeling efficiency and determine changes in overall protein abundance. Peptides with a trypsin-generated α-amine were further captured by reaction with an aldehyde-functionalized high molecular weight polymer and removed by filtration, whereas *in vitro* dimethylated or endogenously N-terminally modified proteins were enriched in the flow-through (27). Mass spectrometric analysis of the preTAILS showed efficient dimethyl labeling (>98%) and identified 371 proteins (Fig. 1*B*), including 271 proteins with annotated mitochondrial location (Fig. 1*C*, supplemental Table S1) as retrieved from the UniProt database (36). A total of 1558 N-terminal peptides from 322 proteins were identified after enrichment by TAILS (Fig. 1*A*, supplemental Table S2). 1058 N-terminal peptides were derived from 214 proteins with UniProt-annotated known or predicted mitochondrial localization (Fig. 1*B*, supplemental Table S3). The biological replicates were highly reproducible, with 775 N-terminal peptides identified in all three experiments and further 334 N-terminal peptides in 2 of the 3 experiments (supplemental Fig. S1A). Overall, a large overlap with our previous quantitative heart proteome analysis was observed (Fig. 1*B*, 1*C*) (13). Next, we retrieved position-specific annotations for each identified N-terminal peptide, including information on protein domain structure and function for the corresponding protein models from UniProt. 262 N-terminal peptides mapped to ”expected” positions, which we here define as N termini starting at positions 1 or 2 of the protein models or within 5 amino acids of known or predicted signal peptide (SP), mitochondrial targeting signal (MTS) or propeptide cleavage sites (Fig. 1*D*). A majority of 1296 N-terminal peptides from 248 proteins mapped to unexpected sites within the predicted protein sequences, representing N termini of unknown origin that likely are mostly protease-generated neo-N termini (Fig. 1*D*). These include peptides from short-lived degradation intermediates but may also indicate processed proteoforms with unknown physiological function, unannotated MTS cleavage sites or proteoforms arising from alternative splicing or use of alternative translation initiation sites (19, 37). Most of the protein N termini mapping to positions 1 and 2 or annotated as secreted peptide (SP) cleavage sites were derived from contaminating non-mitochondrial proteins and were excluded (Fig. 1*D*).

##### Characteristics of the Mitochondrial Mouse Heart N Terminome

**Fig. 2:**
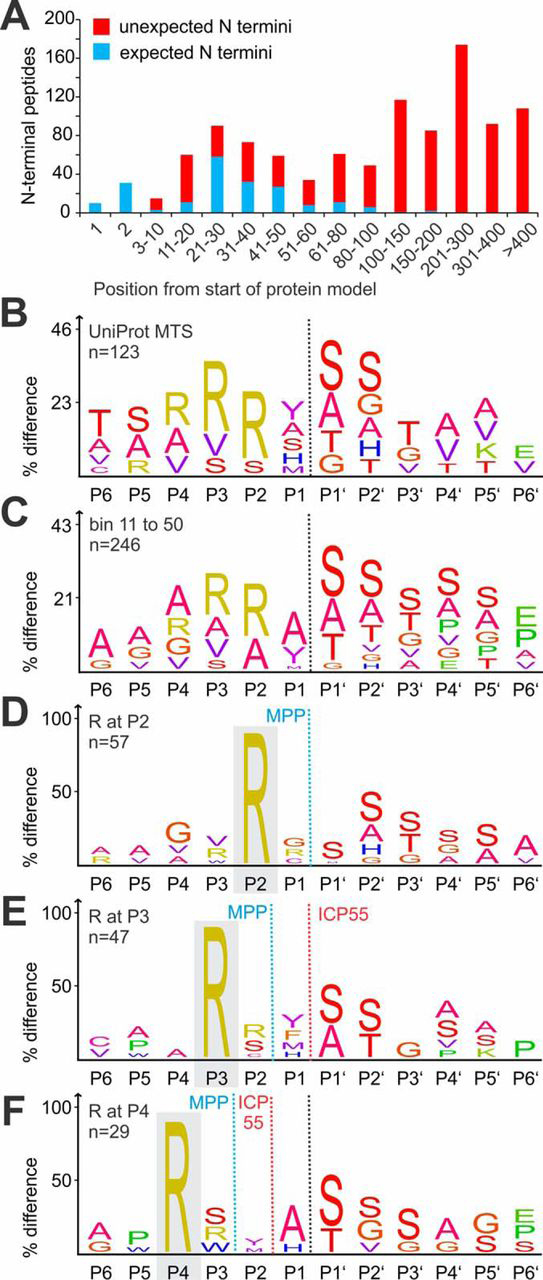
**Analysis of the mitochondrial mouse heart N terminome.** A, Start position of the 1058 identified N-terminal peptides in relation to the corresponding protein model. Blue indicates expected termini mapping to positions 1 or 2 or within 5 residues from a UniProt-annotated mitochondrial targeting signal (MTS) cleavage site, not annotated unexpected N termini are shown in red. IceLogos show amino acids overrepresented in (*B*) 123 unique cleavage sites matching within 5 aa of annotated MTS cleavage sites and (*C*) 246 unique cleavage sites derived from peptides mapping to positions >10 and <51. Further iceLogos visualize amino acids overrepresented among (*D*) 57 cleavages sites with Arg at P2, (*E*) 47 cleavage sites with Arg at P3, and (*F*) 29 cleavage sites with Arg at P4. The dashed black line indicates start of experimentally determined N termini, dashed blue line indicates putative MPP cleavage site, dashed red line putative ICP55 cleavage site.

We then limited further analysis to the 1058 N-terminal peptides matching 963 unique N termini in 214 proteins with UniProt-annotated mitochondrial location (supplemental Table S3). Notably, 172 and 93 of these N termini were previously found by Calvo *et al.* in mitochondria isolated from mouse liver and kidney, respectively (20). Most nuclear encoded proteins targeted to the mitochondria carry an MTS that is cleaved off by the mitochondrial processing peptidase (MPP) after import (38). Hence it was not surprising that 123 non-redundant N termini were observed within 5 amino acid (aa) distance from UniProt-annotated MTS cleavage sites (supplemental Fig. S2A). Many proteins showed N-terminal ragging, *i.e.* sequential truncation by 1 or more amino acids, indicating further aminopeptidase-mediated processing (supplemental Fig. S2A, supplemental Table S3). This is frequently observed in many different compartments and biological systems (17, 30, 39). However, in many cases the most prevalent N terminus can be determined by spectral counts (20), as for example the N terminus starting at Thr26 for ornithine aminotransferase (OAT) (Supplemental Fig. S2B). We further analyzed the position of the N-terminal peptides in relation to the protein model (Fig. 2*A*). As expected, most N termini mapping within 5 amino acids of MTS cleavage sites were observed in the bins between 10 to 50 amino acids from the start (20, 40). Visualization of the cleavage site sequence derived from the 123 N termini mapping close to UniProt-annotated MTS cleavage site revealed an overrepresentation of Arg at P3 and P2 (Fig. 2*B*), reminiscent of the well-described “twin Arg motif” arising from the preferred MTS cleavage with Arg at P2 and subsequent processing by aminopeptidase ICP55-mediated cleavage (20, 25, 41, 42). However, the additional overrepresentation of Arg at P4 indicated further aminopeptidase activity. A similar pattern was observed in an iceLogo analysis of the 246 non-redundant cleavage sites derived from all N-terminal peptides in bins 11 to 50 (Fig. 2*C*), including MTS cleavage sites not yet annotated in UniProt, as for example MRPS22 (supplemental Fig. S2B). Further iceLogo analysis of the 57 N termini with Arg at P2 showed the typical characteristics of MTS cleavage resulting in stable N-terminal peptides frequently starting with Ser (Fig. 2*D*), whereas the 47 N termini with Arg at P3 additionally showed an overrepresentation of aromatic residues Phe and Tyr at P1 (Fig. 2*A*) in agreement with the well-described cleavage site preference of ICP55 for Phe, Tyr and Leu (20, 23, 41, 42, 43). In many cases, protein N termini were frequently further truncated by an unidentified aminopeptidase after MMP and/or ICP55-cleavage. However, neither iceLogo analysis of the 29 cleavage sites with Arg at P4 (Fig. 2*F*) nor inspection of individual examples for well-supported ragged protein N termini (supplemental Fig. S2B) revealed stringent sequence features for this aminopeptidase activity.

Overall, the most N-terminal positions of expected N termini within 5 residues of the known or predicted UniProt-annotated MTS were almost exclusively occupied by Ala (24.6%), Ser (23.0%), Gly (12%), Thr (10.4%) and Met (8.7%) (supplemental Fig. S3), which are all amino acids classified as stabilizing according to the bacterial N-end rule (44). In contrast, protease-generated neo-N termini mapping to positions within the proteins' sequence featured branched aliphatic residues Ile (4.2%), Val (5.7%) and the secondary destabilizing Asp (10.3%) and Glu (5.7%) that were rarely present at this position in expected N termini (supplemental Fig. S3). This supports the hypothesis that a significant proportion of the identified unexpected N-terminal peptides may represent degradation intermediates, which might be recognized by a yet unidentified N-recognin.

##### Neo-N termini Reveal Changes in Protein Processing in CLPP-deficient Heart Mitochondria

**Fig. 3:**
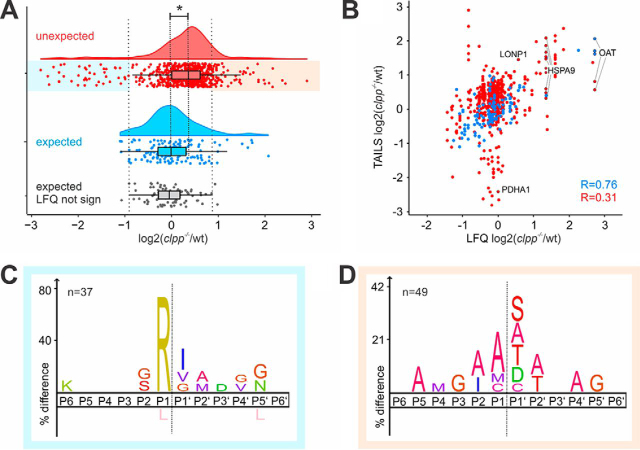
**Quantitative analysis of N termini abundance in *Clpp*^−/−^ and wt mouse heart mitochondria.***A*, Abundance in *Clpp*^−/−^ and wt for 154 expected N-terminal peptides matching within 5 amino acids distance from UniProt annotated translation start or maturation sites and for 623 N-terminal peptides matching to unexpected positions with the corresponding protein model. Asterisk indicates significant difference between the mean values of the two distributions (Mann-Whitney *U* test p-val <0.001), emphasized by dashed line. Red, unexpected N termini; blue, expected N termini; gray, subset of 93 unexpected N termini matching to proteins that showed no significant change in abundance in the LFQ data set used to define cut-off values of significant accumulation (light orange) or depletion (light blue) of unexpected N termini. *B*, Abundance of N-terminal peptides compared with the corresponding protein abundance determined by label-free quantification. Red, unexpected N termini; blue, expected N termini. Pearson correlation for each group is indicated. *C*, iceLogo of 37 unexpected N termini with reduced abundance in CLPP-deficient mitochondria (log_2_(*Clpp*^−/−^/wt)<0.9). *D*, iceLogo of 49 unexpected N termini accumulating in CLPP-deficient mitochondria (log_2_(*Clpp*^−/−^/wt>0.9).

Next we wished to determine differences in N termini abundance between CLPP-deficient and wt heart mitochondria. To account for variations in the amount of co-purified cytosolic proteins in the mitochondrial preparations, we first normalized the ratios in each experiment to the median fold change of the 200 N-terminal peptides matching to expected positions in mitochondrial proteins (Fig. 1*D*, P1/2 or MTS of mitochondrial proteins). We then further restricted the analysis to the 777 N-terminal peptides quantified in at least 2 of the 3 biological replicates (supplemental Table S3), which showed very good reproducibility for paired single peptide data (supplemental Fig. S4A). The median ratio of the 154 expected N-terminal peptides showed the expected symmetric distribution centered around the expected 1:1 ratio (Fig. 3*A*; log_2_(*Clpp*^−/−^/wt) = 0). In contrast, the distribution of 623 unexpected N-terminal peptides was moderately, but significantly shifted (Mann-Whitney *U* test p-val <0.001) to about 25% higher abundance in CLPP-deficient hearts (Fig. 3*A*; Δmedian log_2_(*Clpp*^−/−^/wt) unexpected - expected = 0.32). To further investigate this striking difference, we next compared the N termini abundance with the corresponding protein abundance determined in our previous LFQ proteome (13), as this data set provided quantitative information for more proteins identified by TAILS than our limited preTAILS data set (Fig. 1*C*). The abundance ratio of the 198 proteins quantified in both preTAILS and LFQ data sets correlated well (Pearson correlation 0.73, -lg(p-val)>15.3), demonstrating that the two independent experiments with mitochondria isolated form different mouse cohorts were indeed comparable (supplemental Fig. S4B). In total, 636 of the 777 quantified TAILS-enriched N-terminal peptides matched to 163 proteins quantified in the LFQ data set, of which 165 N-terminal peptides came from 46 proteins with significantly different abundance (Student′s *t* test p-val <0.05). The 120 expected mitochondrial N-terminal peptides correlated well with the overall protein abundance (Fig. 3*B*, Pearson correlation = 0.76, -lg(p-val)>323). This suggested that the known protein N termini largely reflect overall abundance of the corresponding proteins, which further suggests that these constitute the major proteoforms. In contrast, the 389 unexpected protein neo-N termini showed divergent accumulation and reduction and correlated only poorly with the corresponding protein abundance (Fig. 3*B*, Pearson correlation 0.31, -lg(p-val) = 12.3), indicating widespread changes in mitochondrial protein processing in the absence of CLPP. Furthermore, most neo-N termini poorly correlating with protein abundance came from proteins that were not significantly affected by CLPP-deficiency, indicating the cleavages affected only a minor fraction of the corresponding precursor proteins, consistent with short-lived degradation intermediates. Nevertheless, proteolytically generated proteoforms that acquired a new function can be physiologically highly relevant even if only a fraction of the precursor protein is processed (37).

##### Differentially Accumulating N Termini Reveal Candidate ClpXP Substrates

There are many potential direct and indirect causes for differential accumulation of both expected and protease-generated neo-N termini in the absence of CLPP. For example, expected N termini of direct ClpXP substrates would be expected to accumulate with the stabilized protein in the absence of CLPP, whereas the N-terminal peptides derived from CLPP cleavage would be drastically reduced in abundance. However, a plethora of indirect effects are likely to alter the behavior of protein abundance and processing patterns *in vivo*, for example by changes in transcription and translation or differential activation of proteolytic activities that cut the same substrates at the same or different sites. Nevertheless, we reasoned that at least some direct ClpXP substrates, such as stabilized proteoforms with N-terminal ClpXP degrons and N termini generated by ClpXP cleavages, would show strong accumulation in the knockout or wild type, respectively, compared with the 93 expected N-terminal peptides from proteins showing no significant change in abundance in our previous analysis (13). Based on a boxplot analysis of these 93 unchanged termini (Fig. 3*A*), we chose log-transformed ratios ± 0.9, or more than ± 1.5 times the interquartile range, as cut-off values for determining N-terminal peptides with significantly altered abundance (irrespective of protein abundance). This revealed 10 expected and 49 unexpected N termini that were strongly accumulating in CLPP-deficient heart mitochondria, and 6 expected and 37 unexpected N termini strongly reduced in abundance. We speculated that the unexpected N-terminal peptides accumulating in the absence of CLPP might reveal an N-terminal degron for substrate recognition by ClpXP. However, sequence logo analysis of these N termini showed an overrepresentation of the stabilizing residues Ala, Ser and Thr and the potentially destabilizing Asp and Cys as N-terminal residues (Fig. 3*C*), reminiscent of the amino acid abundance observed for expected protein termini (supplemental Fig. S3). In contrast, the 37 neo-N termini that were more prevalent in wt, the best candidates for direct CLPP-mediated cleavage events, showed a clear overrepresentation of Arg at P1 preceding the cleavage site (Fig. 3*D*).

**Fig. 4:**
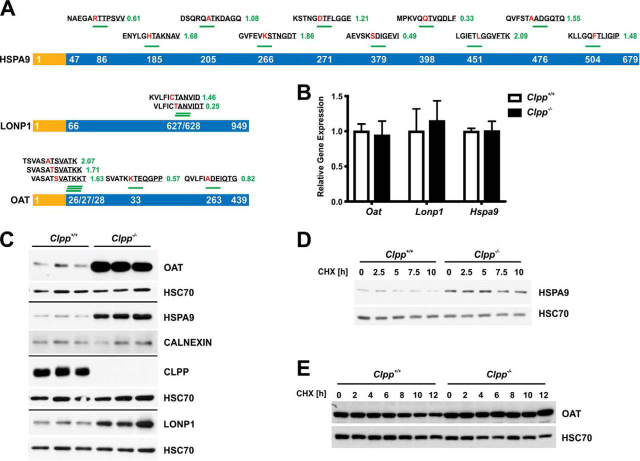
**Validation of candidate ClpXP substrates identified by TAILS.***A*, Putative ClpXP substrates with increased N termini abundance. MTS (yellow) and mature proteins (blue) are shown with the starting positions of the accumulating N termini indicated by white numbers. Above the proteins, cleavage windows are stated with the difference in abundance (log_2_(*Clpp*^−/−^/wt)). Amino acids at the P1 position preceding the cleavage site are highlighted in red, the detected peptide sequence is underlined. *B*, Western blots of steady state protein levels in heart lysates. HSC70 and CALNEXIN were used as loading controls for the respective blots. *C*, Relative gene expression with qPCR of *Oat*, *Lonp1* and *Hspa9. D*, CHX chase experiment of HSPA9 in MEFs. HSC70 was used as loading control. *E*, CHX chase experiment of OAT in MEFs. HSC70 was used as loading control.

One of the proteins with strong differential accumulation of N-terminal peptides was OAT (Ornithine Aminotransferase), where three ragged expected N termini mapping to the known MTS cleavage site accumulated ∼4-fold, in agreement with the protein accumulation observed previously (LFQ log_2_(*Clpp*^−/−^/wt) = 2.71) (13). Notably, two additional protease-generated N termini were much less affected and therefore likely represent intermediates from CLPP-independent degradation (Fig. 3*B*, Fig. 4*A*). We also detected increased abundance of N termini from other proteins that showed significantly elevated protein abundance in our previous study of the *Clpp*^−/−^ heart mitoproteome, including HSPA9 (LFQ log_2_(*Clpp*^−/−^/wt) = 1.34) and LONP1 (LFQ log_2_(*Clpp*^−/−^/wt) = 0.57) (Fig. 3*B*, Fig. 4*A*) (13). Neo-N termini of these proteins were distributed across the protein sequences and the identified cleavage sites differed strongly from those accumulating in the wild type, in line with the overall motifs (Fig. 3*C*, 3*D*). Thus, the detected accumulating neo-N termini are likely the result of alternative quality control proteolysis that is activated in the absence of ClpXP-mediated substrate regulation. Immunoblot analysis confirmed that these proteins (OAT, HSPA9 and LONP1) accumulated in CLPP-deficient heart lysates (Fig. 4*B*), although transcript levels were unchanged (Fig. 4*C*). We further tested if they are indeed bona fide ClpXP substrates by cycloheximide (CHX) chase experiments in cell culture, where cytoplasmic protein synthesis is blocked by CHX treatment and degradation of selected proteins followed over time. As expected, the amount of both HSPA9 (Fig. 4*D*) and OAT (Fig. 4*E*) decreased over time in wild type cells after CHX treatment. In contrast, both proteins were stabilized in CLPP-deficient cells (Fig. 4*D* and 4*E*), although this was less clear for HSPA9 because of the increased overall abundance (Fig. 4*D*). We conclude that OAT and likely also HSPA9 are substrates of ClpXP in murine mitochondria, which is further supported by their interaction with ClpXP in *Podospora anserina* (45). In contrast, LONP1 was not consistently stabilized in CLPP-deficient cells in independent CHX chase experiments (data not shown). At present, we can therefore not rule out that other factors determine LONP1 lifetime, particularly because LONP1 itself is a stress-induced protease activated by the impaired mitochondrial proteostasis (46, 47).

Although we did not observe many examples of N-terminal protein processing that were absent in *Clpp*^−/−^ heart mitochondria, a strongly diminished terminus (log_2_(*Clpp*^−/−^/wt) = −2.41) 15 amino acids after the mitochondrial targeting signal (MTS) cleavage site of PDHA1 (Fig. 3*B*, supplemental Fig. S5A) represented a possible proteolytic modification that is virtually indistinguishable from the full-length protein when analyzed on standard SDS-PAGE (supplemental Fig. S5B) or in the LFQ proteome data set. The pyruvate dehydrogenase is a large complex within the mitochondrial matrix and regulates the oxidative decarboxylation of pyruvate to generate Acetyl-CoA, the entry metabolite of the TCA cycle. Its short-term regulation is mediated through the phosphorylation of the alpha subunit encoded by the *Pdha1* gene (48). Although pyruvate dehydrogenase (PDH) activity in the heart was not affected by CLPP-depletion (supplemental Fig. S5C), it is possible that the truncated PDHA1 protein has altered phosphorylation properties and its processing provides an additional layer of regulation.

##### Identification of Putative ClpXP Substrates by Substrate Trapping

**Fig. 5:**
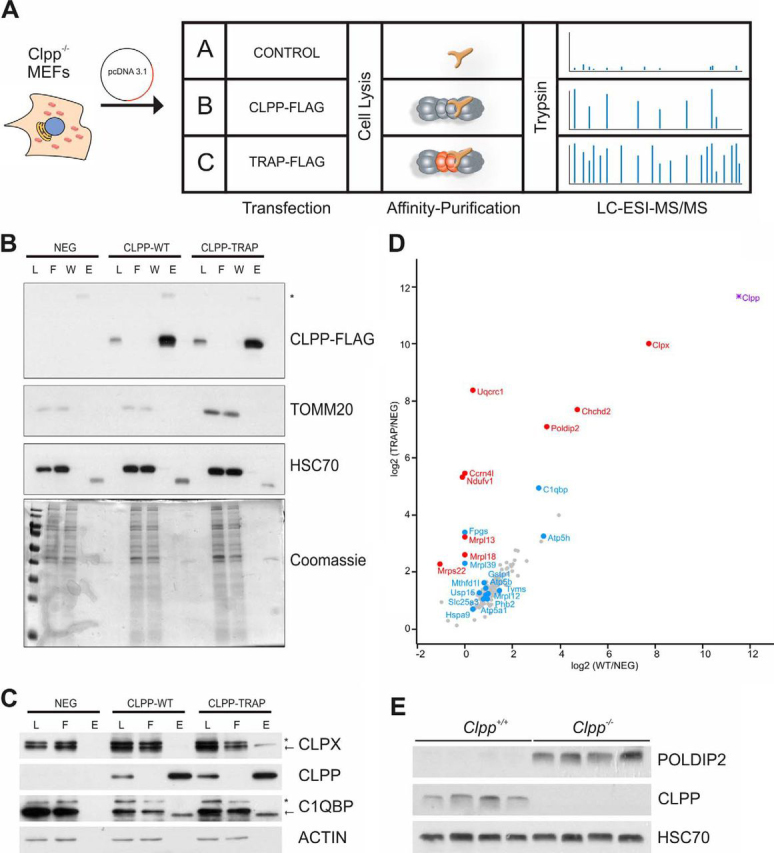
**Identification of candidate ClpXP substrates by trapping.***A*, Trapping workflow: Control, CLPP-WT and CLPP-TRAP containing plasmids were transfected into *Clpp*^−/−^ MEFs. Cells were lysed and FLAG-tagged CLPP was affinity purified with magnetic beads. CLPP and bound proteins were eluted from the beads, subjected to trypsin digestion and quantified with LC-MS. *B*, Western blotting and Coomassie-stained gel of total lysate (L), flow-through (F), washing (W) and elution (E) fractions. TOMM20 and HSC70 were used as controls for unspecific mitochondrial and cytosolic contaminations, respectively. *C*, Western blotting of total lysate (L), flow-through (F) and elution (E) fractions. CLPX and C1QBP/P32 were used as positive controls for proteins known to interact with Clp(X)P. * represents unspecific antibody binding. *D*, Scatter plot of proteins co-enrichment in immunoprecipitates of FLAG-CLPP-TRAP and FLAG-CLPP-WT compared with control. Red dots indicate significantly enriched in CLPP-TRAP over CLPP-WT, blue dots proteins significantly enriched with CLPP-TRAP over negative control. The tight binding ATPase subunit CLPX is highlighted in violet. *E*, Western blotting of POLDIP2 steady state levels in heart lysates, HSC70 was used as control.

Previously, we reported several ClpXP substrates that were initially detected in a substrate screen based on the affinity purification of wild type and catalytically inactive CLPP variants from mitochondria purified from MEFs. We noted that many proteins with altered proteolytic processing patterns were included in our previous substrate trapping screen (13). Although this demonstrated that protease substrates can be co-purified with an active protease, “trapped” substrates are expected to accumulate to higher abundance when purified with an inactive protease that mitigates direct degradation *in vivo* (49). In order to obtain such quantitative data, we repeated the CLPP substrate trapping experiment in triplicate with some modifications to the protocol, including the use of whole cell lysates instead of isolated mitochondria (Fig. 5*A*). This enabled faster and thus more reproducible isolation of CLPP-associated proteins for the quantitative approach, albeit at the cost of a lower coverage of low abundant mitochondrial proteins. Both CLPP-FLAG constructs were expressed to similar extend in CLPP-deficient MEFs and immunoprecipitation with anti-FLAG magnetic beads yielded virtually complete recovery of CLPP-(TRAP)-FLAG protein and minimal contamination with abundant non-interactors such as HSC70 and TOMM20 (Fig. 5*B*) or ACTIN (Fig. 5*C*). Mass spectrometric analysis of whole lysate pull-downs identified 60 mitochondrial proteins, of which 23 and 12 mitochondrial proteins were significantly more abundant after immunoprecipitation with the CLPP-TRAP mutant and CLPP wild type, respectively, compared with the negative control (Multi-sample ANOVA with Benjamini-Hochberg FDR <0.05, followed by Tukey′s PostHoc test with p-val <0.05 to discriminate between the different groups, Fig. 5*D*; supplemental Table S4). As expected and previously observed, the interacting ATPase CLPX (8) was highly enriched in both wild type and inactive IPs, whereas below detection limit in the negative control. Furthermore, C1QBP/P32, a previously suggested ClpXP interactor (10), was also purified with both CLPP variants (Fig. 5*D*, Table III). Interestingly, CLPX resulted in substrate-like co-purification, as it was significantly more enriched with inactive CLPP-TRAP than with wild type CLPP (Fig. 5*D*, Table III). An increased interaction of CLPX and inactive CLPP might arise from trapped substrates anchoring the ATPase to the proteolytic chamber, thus stabilizing the interaction of all three components. Indeed, the stabilization of CLPX with the catalytically inactive CLPP variant also lead to a small but not significant enrichment of the CLPX interactor C1QBP in the CLPP-TRAP fraction (log_2_(TRAP/NEG) = 4.94 *versus* log_2_(WT/NEG) = 3.10). However, because of increased steady-state CLPX levels in *Clpp*^−/−^ mice (13), we cannot rule out that CLPX is also a ClpXP substrate through self-regulating proteolysis. Among the proteins highly enriched with CLPP-TRAP, polymerase delta-interacting protein 2 (POLDIP2) and coiled-coil-helix-coiled-coil-helix domain containing 2 (CHCHD2) were already detected in our initial substrate screen (13). Interestingly, POLDIP2 was recently reported to interact with CLPX to regulate the degradation of ACSM1 (50) and co-purified with human CLPP after crosslinking in HepG2 cells (51). However, we observed an increased amount of POLDIP2 protein in *Clpp*^−/−^ hearts both in our previous proteome analysis (13), and by immunoblotting (Fig. 5*E*), which may indicate a protease-substrate relationship. CHCHD2 recently gained attention as mutations in this gene have been associated with neurodegenerative diseases, including Parkinson's Diseases (52). It was proposed that CHCHD2 competes with OPA1, a dynamin-like GTPase that regulates mitochondrial fusion and cristae structure, for the binding to C1QBP/P32, a previously identified CLPX interactor (53, 54).

**Table III:** High confidence ClpXP substrates significantly enriched in CLPP-TRAP over CLPP-WT

Gene names	TAILS	LFQ log_2_ (*Clpp*^−/−^/WT)	ANOVA q-value	log_2_ (TRAP/WT)	Peptides	Function
*Clpx*	No	6.52	0.00	2.27	30	Known Clp(X)P binding partners
*Chchd2; Zbed5*	No	1.18	0.00	2.98	5	
*Poldip2*	No	2.53	0.00	3.66	18	
*Mrpl13*	No	n.d.	0.00	3.20	6	Mitochondrial Translation
*Mrpl18*	Yes	n.d.	0.01	3.65	2	
*Mrps22*	Yes	1.18	0.02	3.35	6	
*Ndufv1*	Yes	−0.84	0.00	5.45	10	Respiratory Chain
*Uqcrc1*	Yes	−0.13	0.00	8.04	16	
*Ccrn4l*	No	n.d.	0.01	5.27	9	Metabolism

Besides these candidates, further 6 proteins with annotated mitochondrial localization bound significantly stronger to CLPP-TRAP than to active CLPP and were therefore considered as high confidence ClpXP substrates (Table III), whereas additional 12 proteins were, like C1QBP, significantly enriched with inactive CLPP-TRAP over the negative control, suggesting putative ClpXP substrates or interactors (Table IV). Most candidates displayed a similar trend in the initial screen (13), and mainly comprise proteins involved in mitochondrial translation, the respiratory chain and metabolism. Multiple candidates obtained from the substrate screen were also detected in the N terminome data, which largely recapitulated increased steady state protein/degradation intermediate levels in *Clpp*^−/−^ heart mitochondria (supplemental Fig. S6).

**Table IV:** Putative ClpXP substrates and interactors significantly enriched in CLPP-TRAP over NEG

Gene names	TAILS	ANOVA q-value	log_2_ (TRAP/NEG)	log_2_ (WT/NEG)	Peptides	Function
*Clpp*	No	0.00	11.65	11.52	11	Known Clp(X)P binding partners
*C1qbp*	No	0.00	4.95	3.10	5	
*Mrpl12*	No	0.01	1.23	0.94	3	Mitochondrial Translation
*Mrpl39*	Yes	0.02	2.31	0	4	
*Atp5a1*	Yes	0.02	1.06	0.93	14	Respiratory Chain
*Atp5b*	Yes	0.02	1.44	0.89	12	
*Atp5h;Gm10250*	Yes	0.00	3.27	3.30	4	
*Tyms*	No	0.01	1.36	1.43	13	Metabolism
*Mthfd1l*	No	0.00	1.23	0.97	46	
*Fpgs*	No	0.03	3.38	0	3	
*Gstp1*	No	0.03	1.63	0.80	3	
*Slc25a3*	Yes	0.04	1.04	0.76	3	Other
*Usp15*	No	0.01	1.26	0.60	8	
*Phb2*	No	0.00	1.18	0.88	11	

However, two of the proteins with the strongest accumulation in CLPP-TRAP over wild type, the respiratory chain complex I (CI) subunit NDUFV1 and the complex III (CIII) subunit UQCRC1 (Fig. 5*D*, Table III), did not show the intuitively expected increased abundance in *Clpp*^−/−^ heart mitochondria in our previous LFQ data set (13).

##### Confirmation of Complex I N-module Subunits as ClpXP Substrates

**Fig. 6:**
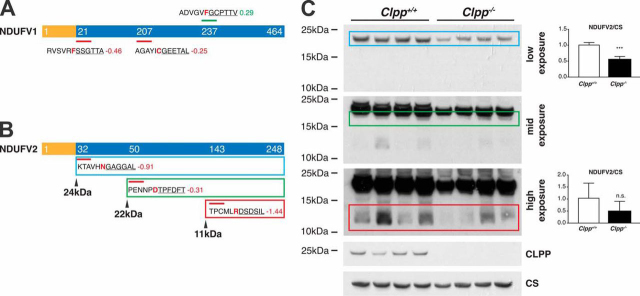
**Altered proteolytic processing of NDUFV1 and NDUFV2 in CLPP-deficient mice.***A*, Scheme of NDUFV1 with identified N termini indicated. MTS (yellow) and mature protein (blue) are shown with starting position of detected N termini. Above and below the protein, identified N-terminal peptides (underlined) are shown with the associated log_2_(*Clpp*^−/−^/wt) and the preceding sequence. Amino acids at the P1 position are highlighted in red. N termini with increased abundance are depicted in green, with decreased abundance in red and unchanged abundance in gray. *B*, Schemes of NDUFV2 with identified N termini using the same color scheme and predicted masses of the corresponding proteoforms. *C*, Immunoblot analysis of NDUFV2 with quantification of the corresponding bands. Differences between wild type and *Clpp*^−/−^ were tested using a two-tailed Student's *t* test. *** indicates p-val <0.001.

We have recently provided strong evidence that NDUFV1, together with the other CI core N-module subunit NDUFV2, is under direct proteolytic control by ClpXP (55). Counterintuitively, NDUFV1, the trapped subunit of CI (Fig. 5*D*) exhibited decreased abundance of the expected N terminus at Ser21 after MTS processing (Fig. 6*A*), in agreement with the reduced protein abundance observed in our previous LFQ proteome analysis (13). NDUFV2 was detected in 2 out of 3 CLPP-TRAP replicates and not in any CLPP-WT or control immunoprecipitations but did not meet our criteria for significance after imputation of missing values for ANOVA analysis (supplemental Table S4). N-terminal peptides of NDUFV2 indicated reduced abundance of the expected MTS-processed N terminus as well as neo-N termini indicating altered degradation (Fig. 6*B*). Immunoblotting confirmed reduced NDUFV2 steady-state levels in CLPP-deficient heart mitochondria and differential accumulation of degradation intermediates (Fig. 6*C*). Together, this data further supports NDUFV1 and NDUFV2 as direct ClpXP substrates, which serves to maintain CI fully functional by enabling selective exchange of N-module components in the pre-existing CI (55).

##### Validation of UQCRC1 As a New ClpXP Substrate

To understand the underlying protease-substrate relation, we further investigated the pattern of UQCRC1-derived N termini identified by the TAILS experiment. Like other abundant proteins, including subunits of the respiratory chain complexes, the CIII subunit UQCRC1 was detected with several distinct N termini (Fig. 7*A*, supplemental Table S3). Whereas the abundance of the expected N terminus after MTS cleavage was not affected in *Clpp*^−/−^, three groups of neo-N termini in close proximity around the 40 aa, the 150 aa, and the 190 aa position showed increased levels in *Clpp*^−/−^ mitochondria. On the other hand, 5 distinct neo-N termini further toward the C terminus of the protein had decreased levels in the knockout (Fig. 7*A*). This pattern suggests that UQCRC1 is initially processed by an unknown protease; a process unaffected by the loss of CLPP and consistent with the unaltered steady-state levels in the label-free proteome analysis of *Clpp*^−/−^ heart mitochondria (13). The released truncated proteoforms of UQCRC1 (30–35 kDa) appear to contain degrons that are recognized by ClpXP, as the loss of CLPP increased the abundance of N termini of the truncated protein and decreased those produced in the presence of ClpXP. In agreement with this interpretation, we observed the accumulation of a truncated form of UQCRC1 in *Clpp*^−/−^ mitochondria by immunoblotting (Fig. 7*B*) and an increased UQCRC1 half-life in cycloheximide-chase experiments (Fig. 7*C*). Together these results suggest that UQCRC1 stability and abundance is regulated by a proteolytic cascade that involves final degradation by the ClpXP machinery.

## DISCUSSION

In bacteria, substrate recognition, selectivity and timing of degradation by the caseinolytic protease ClpP are regulated by its interactions with different AAA^+^ ATPase subunits and adapter molecules (56). For example, arginine phosphorylation marks substrates for degradation by ClpAP in *Bacillus subtilis* (57), whereas ClpXP recognizes and degrades *E. coli* proteins marked with specific N- and C-terminal sequences (9). In contrast, it is not clear how specific proteins are marked for selective degradation by ClpXP in mitochondria.

Our N-degradomic analysis of mouse heart mitochondria identified 110 expected N termini mapping at or close to the predicted MTS cleavage sites, which showed good agreement with previously reported cleavage site characteristics and N-terminal amino acid prevalence (20, 25, 42). Mapping of the 1058 N-terminal peptides from mitochondrial proteins to positions within the respective protein models suggested that a few more N termini may be derived from MTS processing and subsequent maturation, but the vast majority indicated proteolytic cleavage often at multiple sites within the target proteins. These protease-generated neo-N termini showed an increased frequency of Ile, Thr, Glu and Asp as the most N-terminal residue compared with the expected N termini, where Met, Ala and Ser were more prevalent. Surprisingly, protease-generated neo-N termini exhibited a shift to higher abundance in CLPP-deficient mitochondria, indicating that CLPP directly or indirectly affects general proteostasis pathways. Two of the amino acids that are more prevalent in the protease-generated N termini, Thr and Asn, have been observed as part of an N-terminal ClpXP degron in *E. coli* (9), whereas Asp and Asn are also secondary destabilizing residues controlled by the N-end rule pathway in some bacteria (44). Similar N-degron pathways involving ClpP-mediated proteolysis operate in the chloroplasts of higher plants (58, 59, 60). It is thus tempting to speculate that the observed differences in N-terminal amino acid frequency may reflect similar N-terminal degrons for mitochondrial ClpXP substrate targeting. However, this hypothesis requires further experimental testing and clarification if adaptor subunits are indeed involved in the ClpXP substrate recognition in mitochondria.

Using our combined biochemical and mass spectrometry approach, we identified several new bona fide ClpXP substrates that were validated in biochemical and cell culture assays. In line with our previous observations, ClpXP appears to regulate the abundance of selected mitochondrial proteins at key positions in various metabolic pathways. Similar to ERAL1 with regard to mitoribosome maturation (13), or VLCAD in mitochondrial fatty acid oxidation (11), we suggest that ClpXP might regulate processes such as iron-sulfur or amino acid biosynthesis through proteolytic control of HSPA9 and OAT abundance, respectively. Such selective regulation of the abundance of key proteins in important biosynthetic pathways is an efficient and rapid way to adapt mitochondrial function and homeostasis to varying conditions. Although LONP1 protein levels increase in the absence of CLPP and LONP1 interacted with inactive CLPP in our initial trapping screen (13), the protein was not consistently stabilized in *Clpp*^−/−^ MEFs. *E. coli* Lon protease is a known ClpXP substrate (9) and mammalian LONP1 and ClpXP have been suggested to interact under depolarizing conditions (61), proposing that ClpXP also regulates LONP1 in mammalian mitochondria. We have previously observed that CLPX abundance is strongly increased in CLPP-deficient hearts and MEFs whereas the transcript levels were unaffected (13). Here, we found that the interaction of CLPX with catalytically inactive CLPP is stabilized compared with wild type CLPP. Thus, we suggest that mammalian ClpXP possesses intrinsic self-regulation, which was also reported for bacterial ClpXP (9) and other mitochondrial proteases (62, 63).

Our data indicated that mammalian ClpXP also contributes to the degradation of another class of substrates that do not necessarily accumulate in the absence of the protease, hence could only be identified with interaction studies or degradomic approaches such as TAILS. We have recently shown that NDUFV1 and NDUFV2, two CI N-module subunits that are also identified in the substrate screen here, are ClpXP substrates with decreased abundance in the absence of CLPP (55). As demonstrated here, UQCRC1 is likely another example of such a substrate, and accumulation of a processed substrate suggested that its proteolysis is regulated by two or more proteases, one of which is ClpXP. This suggests that the activities of various proteolytic enzymes in mitochondria are interdependent, similar to the mammalian protease network in the cytoplasm (64). In *E. coli*, ClpXP is primarily a regulatory protease that might also contribute to basic housekeeping functions in protein quality control, including the degradation of partially folded substrates that cannot be unfolded and degraded by the Lon protease (56). It is therefore possible that the observed cooperative degradation of UQCRC1 is of quality control nature, and that also mitochondrial ClpXP is able to process unstable protein fragments that result from incomplete degradation. On the other hand, truncated proteoforms of UQCRC1 may exert distinct physiological functions that are tightly controlled by ClpXP-mediated degradation. In *Clpp*^−/−^ mitochondria, the abundance of truncated UQCRC1 is much lower than the full-length protein and it remains to be determined whether the presence of these truncated forms can affect CIII integrity or trigger a specific adaptive response when its abundance reaches a certain threshold.

Finally, we speculated that N termini directly generated by CLPP would be highly enriched in the wild type, either as stable fragments or as transient degradation intermediates. Most of these events were observed for proteins that did not significantly change in abundance (Fig. 3*B*), including peptides mapping to the previously validated substrate NDUFV2, and UQCRC1, a ClpXP substrate validated here. Other proteins in this group include various subunits of respiratory chain complexes such as UQCRC2, CYC1, COX4I1 and SDHA (supplemental Table S3) that we consider as attractive substrate candidates for validation in future studies. We further speculated that these neo-N termini, absent in the CLPP-deficient mice, would allow us to define the cleavage specificity of murine CLPP. Indeed, we observed a prominent overrepresentation of Arg at P1 (Fig. 3*C*), which is also reflected in the cleavage sites observed in the validated substrate UQCRC1. In striking contrast, *in vitro* studies with recombinant bacterial and human CLPP rarely observed Arg at the position preceding the cleavage site (65). Therefore, murine ClpXP might differ in cleavage specificity, which must be tested in future experiments with recombinant murine ClpXP. Alternatively, these cleavage sites might be observed mostly *in vivo* because of the slowing of processive ClpXP degradation at these unfavored sites, or differences in substrate selection with yet unidentified mitochondrial adapter proteins.

Taken together, our study suggests a dual function of ClpXP as a regulator of major mitochondrial functions by selective degradation of specific substrates, and as a component of a protease network controlling proteostasis in mitochondria. Yet, it remains to be resolved how ClpXP recognizes its substrates and whether the latter function is exerted directly by contributing to the degradation of partially folded degradation intermediates released, or indirectly by regulating the abundance of LONP1 or other quality control proteases.

## DATA AVAILABILITY

MS data have been deposited to the ProteomeXchange Consortium (http://www.proteomexchange.org) (66) via the PRIDE partner repository (https://www.ebi.ac.uk/pride/archive/) (67) with the identifiers PXD018194 for the preTAILS data set, PXD014159 for the TAILS N terminome data set and PXD015399 for the CLPP substrate trapping data set.
