# Supplementary material for: The Mouse Heart Mitochondria N Terminome Provides Insights into ClpXP-Mediated Proteolysis
Source: Mol Cell Proteomics. 2020 Nov 23;19(8):1330–45. doi: 10.1074/mcp.RA120.002082 (PMC8014998; doi:10.1074/mcp.RA120.002082)
Supplement: Supplementary file 1 [file mmc1.zip › 160029_1_supp_533813_qcmcnq.pdf]

Supplemental material for

The mouse heart mitochondria N terminome provides insights into ClpXP-mediated proteolysis

Eduard Hofsetz[1,2], Fatih Demir[3], Karolina Szczepanowska[1,2], Alexandra Kukat[1,2], Jayachandran N. Kizhakkedathu[4], Aleksandra Trifunovic[1,2,*], Pitter F. Huesgen[2,3,5,*]

[1]Institute for Mitochondrial Diseases and Aging at CECAD Research Centre, and Center for Molecular Medicine Cologne (CMMC), Medical Faculty, University of Cologne, Cologne, Germany;

[2]Cologne Excellence Cluster on Cellular Stress Responses in Aging Associated Diseases (CECAD), Cologne, Germany, Medical Faculty and University Hospital, University of Cologne, Cologne, Germany

[3]Central Institute for Engineering, Electronics and Analytics, ZEA-3, Forschungszentrum Jülich, Germany

[4]Centre for Blood Research, School of Biomedical Engineering, Department of Pathology & Laboratory Medicine, Department of Chemistry, University of British Columbia, Vancouver BC, Canada

[5]Institute for Biochemistry, Faculty of Mathematics and Natural Sciences, University of Cologne, Cologne, Germany

* Correspondence to: Aleksandra Trifunovic (aleksandra.trifunovic@uk-koeln.de) or Pitter F. Huesgen (p.huesgen@fz-juelich)

Supplemental Tables

Supplemental table 1. Mitochondrial proteins identified in the preTAILS dataset. *(provided as separate xls-spreadsheet)*

Supplemental table 2. List of all peptide-sequence-matches for peptides identified in the TAILS dataset. *(provided as separate xls-spreadsheet)*

Supplemental table 3. List of 1058 N-terminal peptides of mitochondrial proteins identified by TAILS. *(provided as separate xls-spreadsheet)*

Supplemental table 4. List of mitochondrial proteins identified after FLAG-immunoprecipitation from cell lysates of CLPP-TRAP-FLAG, CLPP-FLAG and vector control transfected MEF cells. *(provided as separate xls-spreadsheet)*

Supplemental Figures

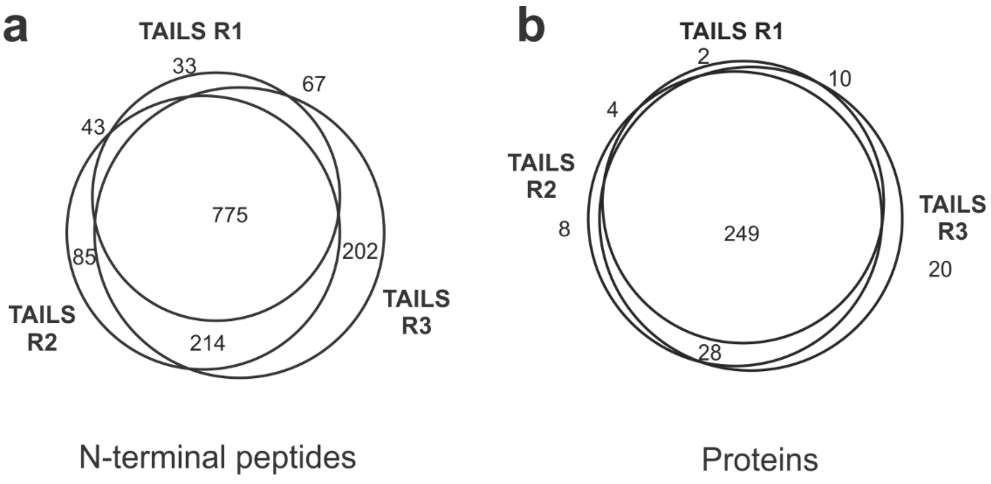

Figure S1. Comparison of the TAILS analysis from three biological replicate experiments. (**a**) Overlap of N-terminal peptides identified in the three replicates. (**b**) Overlap of proteins identified in the three replicates.

a

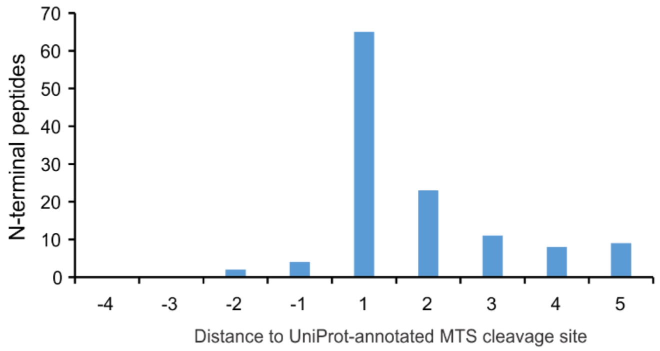

b **OAT** (Q8BFP9), Ornithine aminotransferase

MTS	N-terminal peptide	start	liver	kidney	spectra	proteases
26	MLSKLASLQTIAALRRGVHTSVASATSVATKKTEQGPPSSEYIFER	26	+	+	24	MPP
	SVATKKTEQGPPSSEYIFER	27	+		4	MPP + unknown AP
	VATKKTEQGPPSSEYIFER	28			3	MPP + unknown AP
	ATKKTEQGPPSSEYIFER	29			2	MPP + unknown AP
	TEQGPPSSEYIFER	33			2	MPP + unknown AP

c **MRPS22** (Q9CXW2), 28S ribosomal protein S22

MTS	N-terminal peptide	start	liver	kidney	spectra	proteases
n/a	MAAVRTPLSLWRFQLGSRRA RRVCTRATAQ RHPDALLATR PQPFEVGQPRRLLSSEAESGSSEVKKPAFMDEEVQR	54	+	+	5	MPP + ICP55

d **PDK1** (Q8BFP9), [Pyruvate dehydrogenase (acetyl-transferring)] kinase isozyme 1

MTS	N-terminal peptide	start	liver	kidney	spectra	proteases
26	MRLARLLRGG TSVRPLCAVPCASRSLASASASGSGPASELGVPGQVDFYAR	27	+		23	MPP + ICP55
	SASASGSGPASELGVPGQVDFYAR	28			20	MPP + ICP55 + unknown AP
	ASASGSGPASELGVPGQVDFYAR	29			32	MPP + ICP55 + unknown AP
	SASGSGPASELGVPGQVDFYAR	30			8	MPP + ICP55 + unknown AP
	ASGSGPASELGVPGQVDFYAR	31			7	MPP + ICP55 + unknown AP

Figure S2. Analysis of the mitochondrial mouse heart N terminome. (**a**) Distance of 123 unique N termini mapping to proteins within 5 residues from a UniProt-annotated mitochondrial targeting signal (MTS) cleavage site. Examples show N-terminal peptides indicating (**b**) MPP cleavage with subsequent N-terminal processing by an unknown aminopeptidase in OAT, (**c**) the MTS cleavage site in MRPS22 that is so far not annotated in UniProt, and (**d**) MPP cleavage with subsequent processing by ICP55 and an unidentified aminopeptidase in PDK1. Identified peptides shown in black, preceding putative MTS sequence in light grey. MTS, UniProt-annotated MTS length, liver and kidney signify previous observation by Calvo et al [21]; spectra, MS/MS spectral counts as a proxy of abundance; proteases, putative enzyme activity resulting in the observed N terminus.

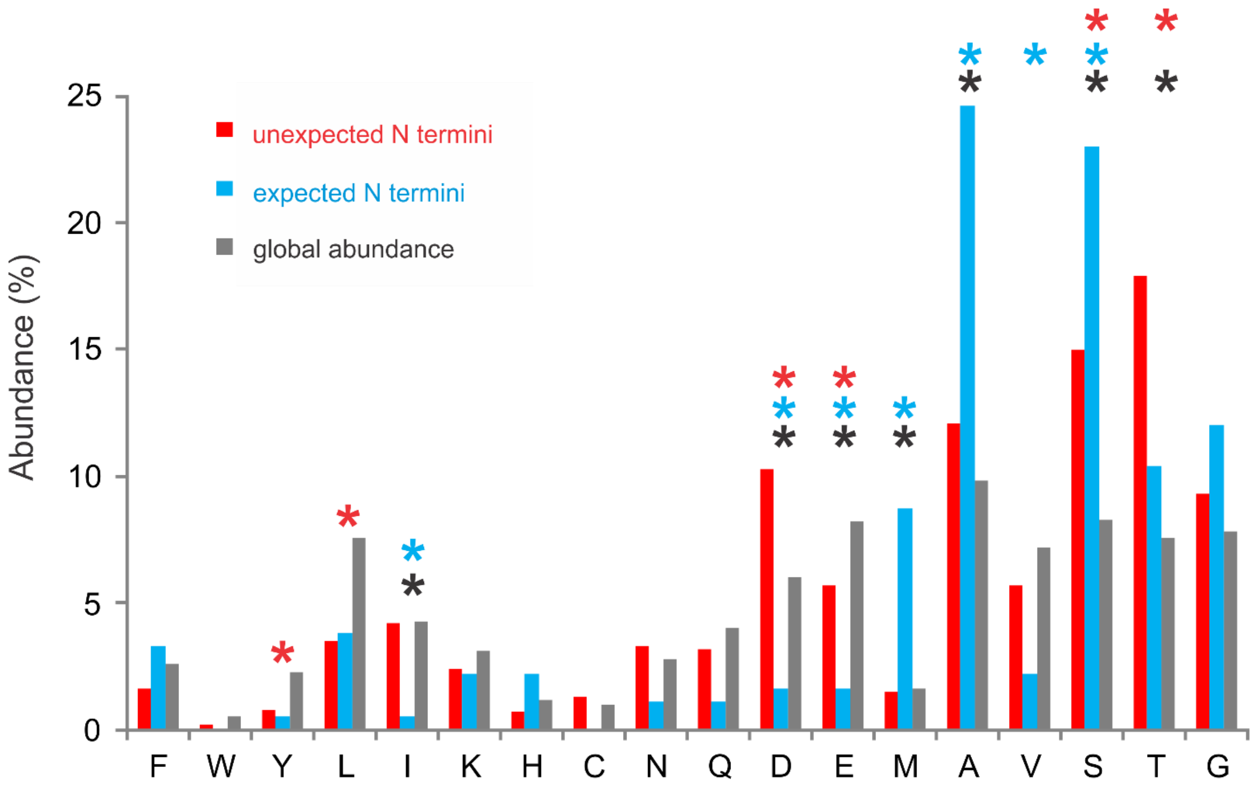

Figure S3. Amino acid occurrence at the most N-terminal position of 1058 N-terminal peptides from mitochondrial proteins. Blue indicates expected termini, red indicates unexpected N termini, and grey shows the overall abundance of each amino acid in the identified N-terminal peptides as a proxy for the natural abundance. Black asterisks indicate significant differences (Fisher´s exact test, p-value <0.05) between the abundance of the most N-terminal amino acid between expected and unexpected N termini. Red and blue asterisks indicate significant differences of the N-terminal amino acid abundance of expected and unexpected N termini, respectively, compared to the overall amino acid abundance.

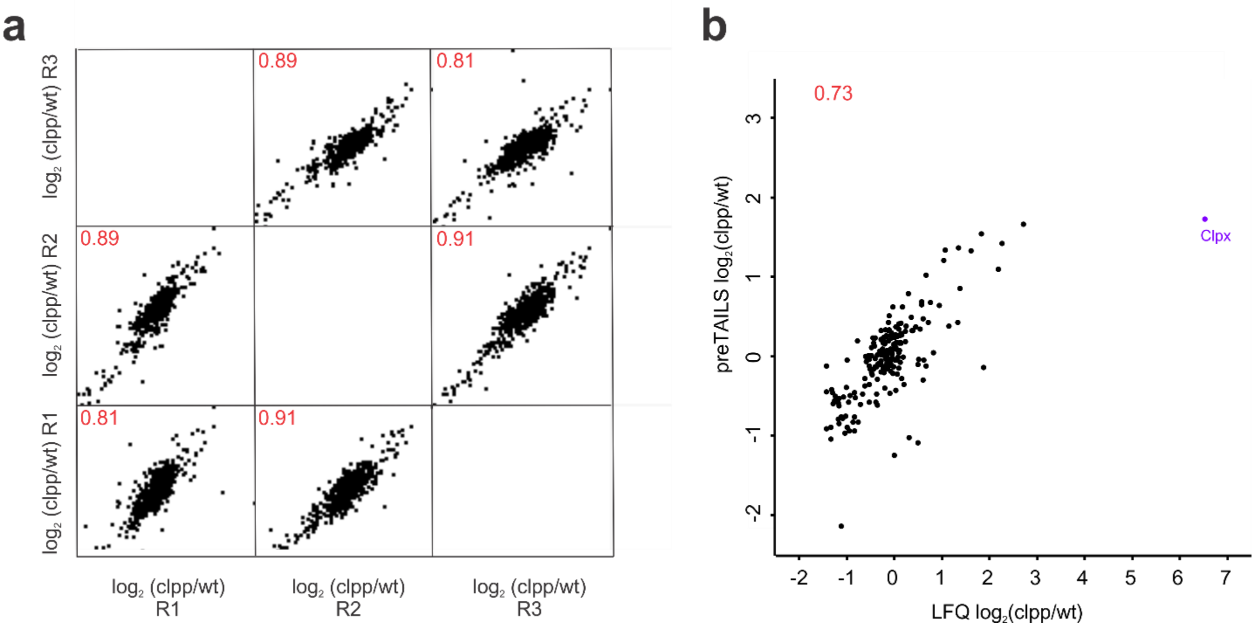

Figure S4. Assessment of N termini and protein quantification (**a**) Reproducibility of N-terminal peptide quantification. Correlations of the $\log_2(Clpp^{-/-}/\text{wt})$ for the 777 N-terminal peptides quantified in at least 2 of the 3 biological replicates are shown and Pearson correlation factors are indicated. (**b**) Correlation of protein abundance data based on 198 proteins quantified in both the preTAILS dataset and our previous LFQ analysis [13].

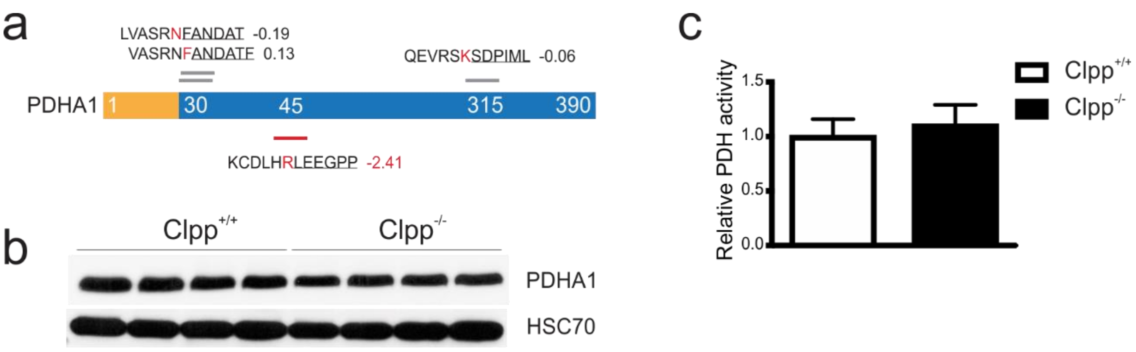

Figure S5. Analysis of PDHA1 as putative ClpXP substrate. (**a**) Abundance and position of N-terminal peptides matching to PDHA1. MTS (yellow) and mature protein (blue) is shown with the position of the identified N-terminal peptides. Decreased abundance is highlighted in red and unchanged abundance in grey. Cleavage windows are stated with the $\log_2$(*Clpp*$^{-/-}$/wt). Amino acids at the P1 position are highlighted in red, the detected peptide is underlined. (**b**) Western blot of PDHA1 steady state levels in heart lysates. HSC70 was used as loading control. (**c**) Colorimetric determination of relative PDH activity in heart lysates.

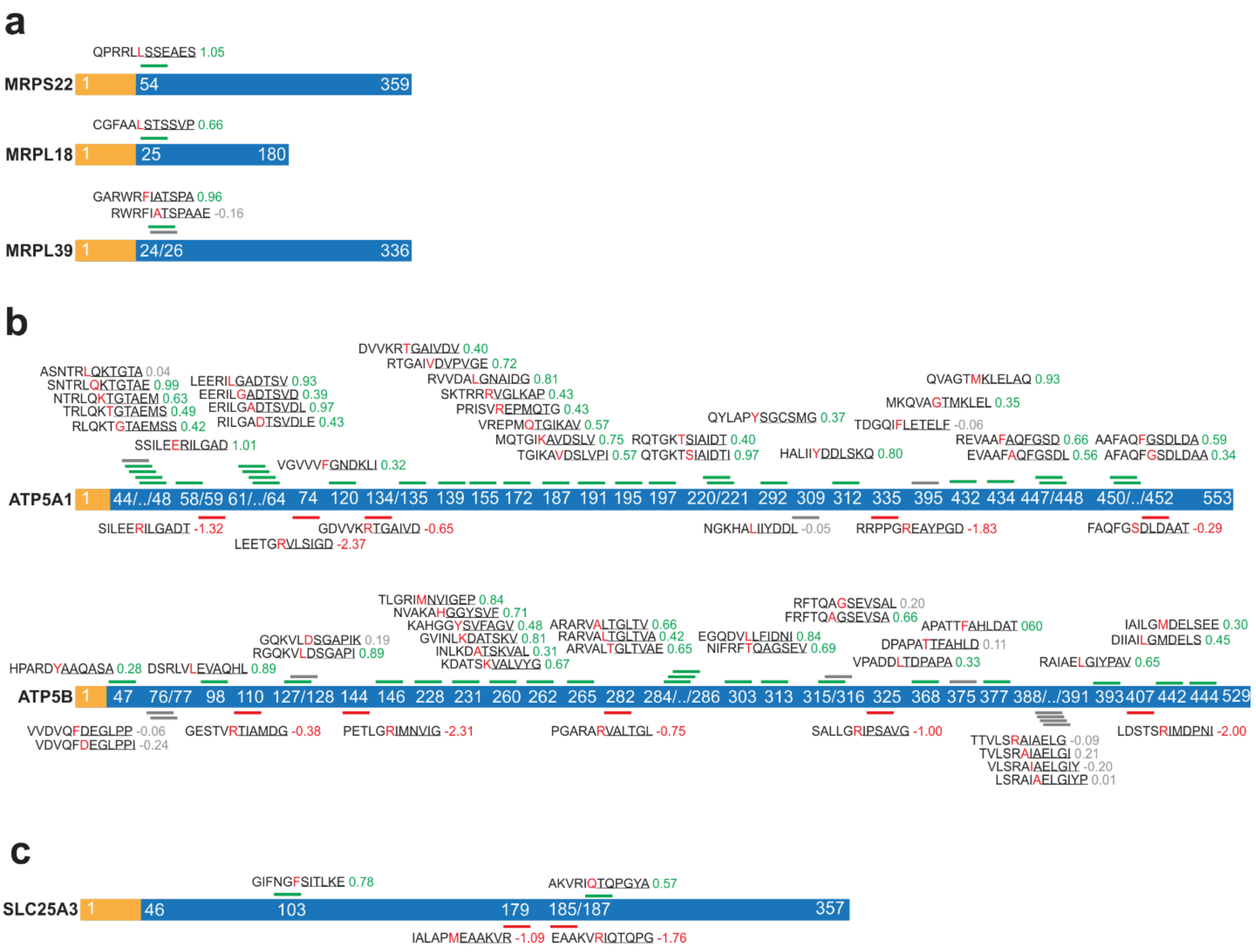

Figure S6. N-terminal peptides from identified substrate/interactor candidates. **(a)** Substrate/interactor candidates involved in mitochondrial translation. (**b**) Substrate/interactor candidates involved in CV. (**c**) Substrate/interactor candidate involved in other functions. N termini abundance. MTS (yellow) and mature proteins (blue) are shown with starting position of elevated N termini. Above the protein, cleavage windows are stated with the $\log_2$(*Clpp*$^{-/-}$/wt). Amino acids at the P1 position are highlighted in red, the detected peptide is underlined. N termini with increased abundance are depicted in green, with decreased abundance in red and unchanged abundance in grey.
